# Edible wild plant species used by different linguistic groups of Kohistan Upper Khyber Pakhtunkhwa (KP), Pakistan

**DOI:** 10.1186/s13002-023-00577-5

**Published:** 2023-02-13

**Authors:** Muhammad Amin, Muhammad Abdul Aziz, Andrea Pieroni, Abdul Nazir, Abdullah Ahmed Al-Ghamdi, Aleyna Kangal, Khalid Ahmad, Arshad Mehmood Abbasi

**Affiliations:** 1 Department of Environmental Sciences, COMSATS University Islamabad, Abbottabad Campus, Abbottabad, 22060 Pakistan; 2 Department of Environmental Sciences, Informatics and Statistics, Ca’ Foscari University of Venice, Via Torino 155, 30172 Venice, Italy; 3 University of Gastronomic Sciences of Pollenzo, Piazza V. Emanuele II, 12042 Bra/Pollenzo, Italy; 4 Department of Medical Analysis, Tishk International University, Kurdistan, Erbil, 4401 Iraq; 5 Department of Botany and Microbiology, College of Science, King Saud University, P.O. 2455, Riyadh, 11451 Saudi Arabia; 6 School of Arts and Sciences, New Brunswick-Piscataway Area Campus of Rutgers University, New Brunswick, USA

**Keywords:** Wild foods, Traditional knowledge, Linguistic groups, Kohistan, Pakistan

## Abstract

**Background:**

The mountainous territory of Kohistan shelters diverse food plant species and is considered one of the important hotspots of local plant knowledge. In the era of globalization and food commodification, wild food plants (WFPs) play an important role in supporting local food systems and related local knowledge is one of the important pillars of food sustainability across the region. Since the area is populated by different cultural groups and each culture has retained particular knowledge on the local plant species, therefore, to make a cross-culturally comparison, the study was planned to record and compare the local plants knowledge among three linguistic groups viz Gujjar, Kohistani and Shina in order to not only protect the local knowledge but to determine the food cultural adaptations among these groups looking through the lens of their food ethnobotanies.

**Methods:**

Field ethnobotanical survey was carried out in 2020–2021 to gather the data on wild food plants. We used semi-structured interviews. Use reports were counted, and the results were visualized through Venn diagrams.

**Results:**

In total, 64 plant species belonging to 45 botanical families were documented. Among these *Ajuga integrifolia*,* Barbarea verna*,* Clematis grata*,* Impatiens edgeworthii*, *Ranunculus laetus* (vegetables),* Parrotiopsis jacquemontiana* (fruit), *Indigofera tinctoria* (flower), *Juniperus excelsa*, *Primula elliptica*, *P. macrophylla* (flavoring agent), *Leontopodium himalayanum* (Chewing gum), and *Juniperus excelsa* (snuff) were reported for the first time. The highest use reports (≥ 90) were recorded for *Mentha longifolia*,* Amaranthus hybridus*,* Quercus semecarpifolia*,* Solanum miniatum*,* Oxalis corniculata*, *Ficus palmata*, and *Urtica dioica*. Maximum number of wild food plant species (WFPs) were reported by Kohistani, followed by Shinaki and Gujjari linguistic groups. The percentage overlap of traditional knowledge on WFPs was highest among Kohistani and Shinaki (56.0%), followed by Shinaki and Gujjars (17.0%), and Kohistani and Gujjars (15.0%). Kohistani and Shinaki groups exhibited maximum homogeneity in traditional knowledge. However, Gujjars had more knowledge on WFPs compared to Kohistani and Shinaki. In addition, some dairy products viz. Bhorus, Bagora, Bak, Cholam, Kacha, Gurloo and Poyeen were reported also reported that are consumed orally and used in traditional cuisines.

**Conclusions:**

The study indicates that Kohistan is one of the important spots of biocultural diversity and could be recognized as *biocultural refugia.* WFPs have been an integral part of the traditional food systems among the studied groups, particularly the Gujjars have reported more distinct plant uses which could be referred to their distinctive ecological experiences among others. However, social change is one of the challenges that might lead to the erosion of local plant knowledge. Moreover, intercultural negotiations among the studied groups are also a matter of concern which could homogenize the local knowledge among them. Therefore, we suggest solid policy measures to protect the local knowledge and celebrate diversity across this mountain territory.

**Supplementary Information:**

The online version contains supplementary material available at 10.1186/s13002-023-00577-5.

## Background

Humans have always assumed adaptive strategies to cope with food insecurity and other environmental hardships since pre-historic times. Seasonal migration among pastoral societies in mountain regions has been a way to sustain livelihood systems, and to better achieve livestock management practices across the world [1–3]. Hence, traditional ecological knowledge of pastoralist communities has been one of the most important components of adaptive and survival approaches both in time and space [1].

Kohistan is the crossroad that connects Central, South, and Southwestern Asia. The area is populated by Dardic people and has a rich cultural history. Geographically, it stretches from Indian Kashmir in the East and expands toward the border of Afghanistan on the Western side. Kohistan is divided into two parts, i.e., eastern, and west sides of the Indus River and splits into three districts viz. Kohistan Upper, Lower Kohistan and Kolai-Palas Kohistan. Mountain pastoralism is frequently practiced among the local communities in the Kohistan region. For instance, Kohistani, Shinaki, and Gujjars are some of the local communities who tend to seasonally move back and forth along with their herds from low land areas of Northwest Frontier Province and Punjab to the high elevation summer pastures of Hindukush and Himalayas. Among all, Gujjars are characterized by their extensive pastoral practices in these highlands, which is one of the cores points to be discussed in this paper and the inferences taken from these findings could better guide future developmental programs across Himalayas, Karakorum, and Hindukush (HKH) regions.

It is important to note that the highlands, especially the alpine meadows, present a vibrant arena for cultural and local ecological activities. The seasonal migration of the pastoral communities provides an opportunity to undergo the different ecological landscapes and restructure their ecological relationships with the surrounding ecosystems. It has been found that in the high alpine regions people use local food plants which become an integral part of their local food systems. Reports have confirmed that plant foragers, herders, and sometimes hunters are primarily involved in gathering and selling food and medicinal plants. For instance, some of the important wild food and medicinal plants such as *Amaranthus hybridus*,* Diospyros lotus*, *Ficus palmata*, *Juglans regia*, *Mentha longifolia*, *Morus nigra*, *Pinus gerardiana*, *Prunus persica*, *Punica granatum*, and *Ziziphus jujuba* are frequently brought to the local markets and sold to the local shopkeepers.

Research studies that explore the foraging of wild food plant species (WFPs) among these high mountain societies and especially the mobile pastoral societies in Asia are very limited. A few studies have been carried out by our research group in different parts of North Pakistan [4–9], and there is still a need for further ethnographic studies to understand the existing patterns of foraging among culturally diverse ethnic groups. It is generally observed that mobile pastoralism provides a good opportunity to plant foragers who can have a better chance to collect plants while practicing their conventional transhumance pastoralism [6]. The ethnobotanical studies devoted to the documentation of local knowledge on WFPs thus should present in-depth discussions to frame policy measures for rural sustainable development in these fragile mountain regions.

It is worth mentioning that the global socio-economic changes have also local impacts and is rapidly altering the local economies of rural societies even in remote mountain areas [10]. For instance, the China–Pakistan Economic Corridor (CPEC) has created job opportunities and led to diversify the local livelihood sources of communities in the mountain regions of the country. The local ecological practices have been constantly changing since the onset of CPEC, and this could be perceived as a challenge to local ecological knowledge as well as local people’s capacities to manage the local ecosystem to produce their own food. Moreover, the social change becomes a huge threat to biocultural diversity too. Therefore, it is crucial to protect the local knowledge systems in order to be able to utilize them in future developmental programs. These programs will serve not only to help the local people, but also to reshape and maintain their sovereign food systems to celebrate the biocultural diversity in these remote mountain areas.

In this attempt, we have focused on recording the foraging practices linked to WFPs among three linguistic groups, i.e., Gujjars, Kohistanis, and Shinaki. Although pastoralism is practiced by all these three groups the traditional lifestyle of Gujjars is particularly characterized by mobile pastoralism which is considered a way of cultural adaptation to the local landscape among them [11–13]. The current study was planned to explore the cross-cultural exchange of local knowledge on wild food resources (while keeping in mind the importance of local foraging practices, and the cultural diversity of the region). In addition, traditional knowledge on making certain unique dairy products was also documented considering that it is an important part of traditional food system of Kohistan.

The specific objectives of the research study were:

1. To record the use of WFPs among the considered linguistic groups.
2. To cross-culturally compare the WFP use among the three linguistic groups and compare with the previous ethnobotanical literature of Pakistan.
3. To make an empirical overview of the traditional food system to provide policy recommendations for future food sovereignty and sustainability.

## Methods

### Study area and communities

**Fig. 1:**
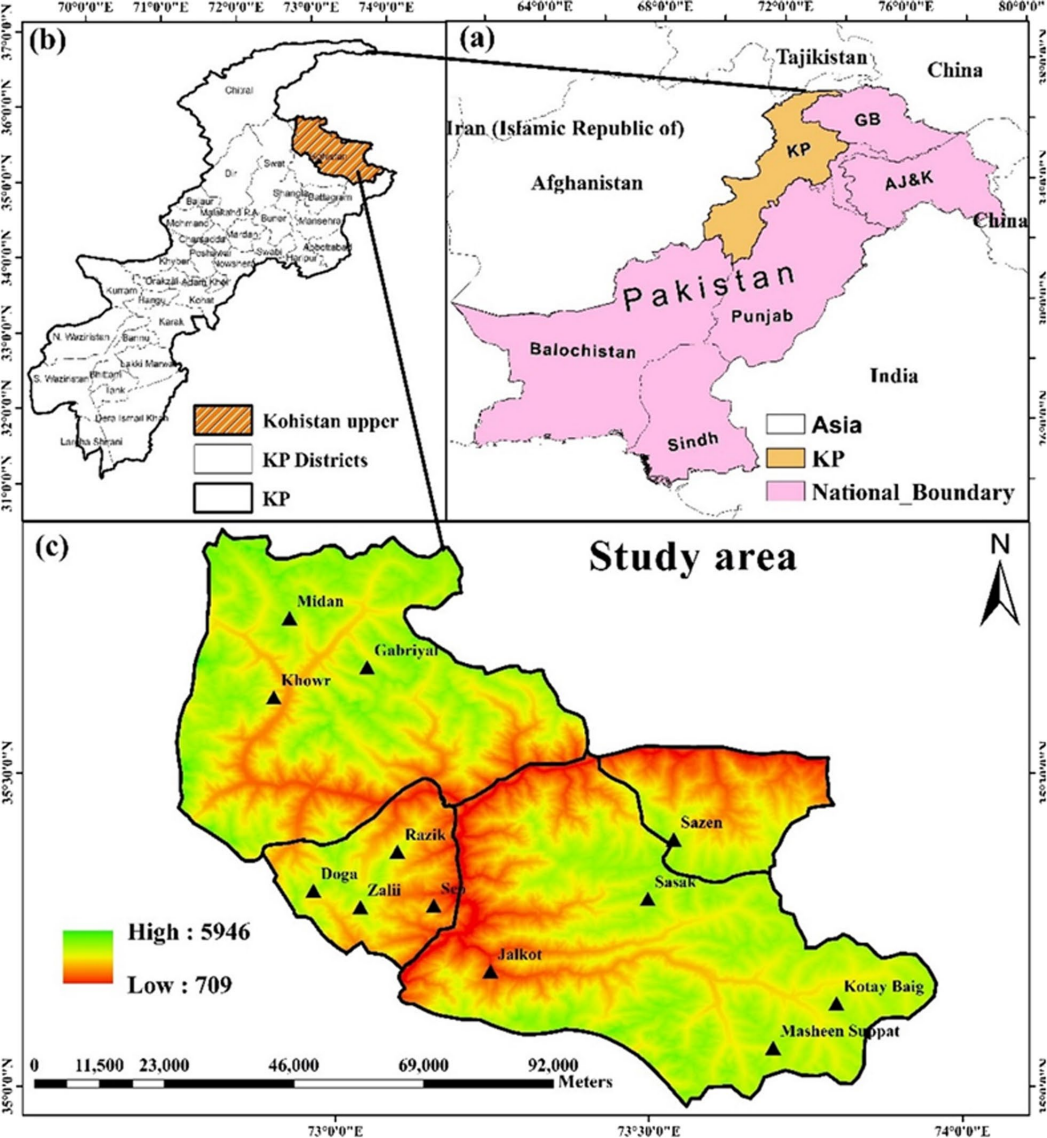
Map of the study area showing sampling sites

**Fig. 2:**
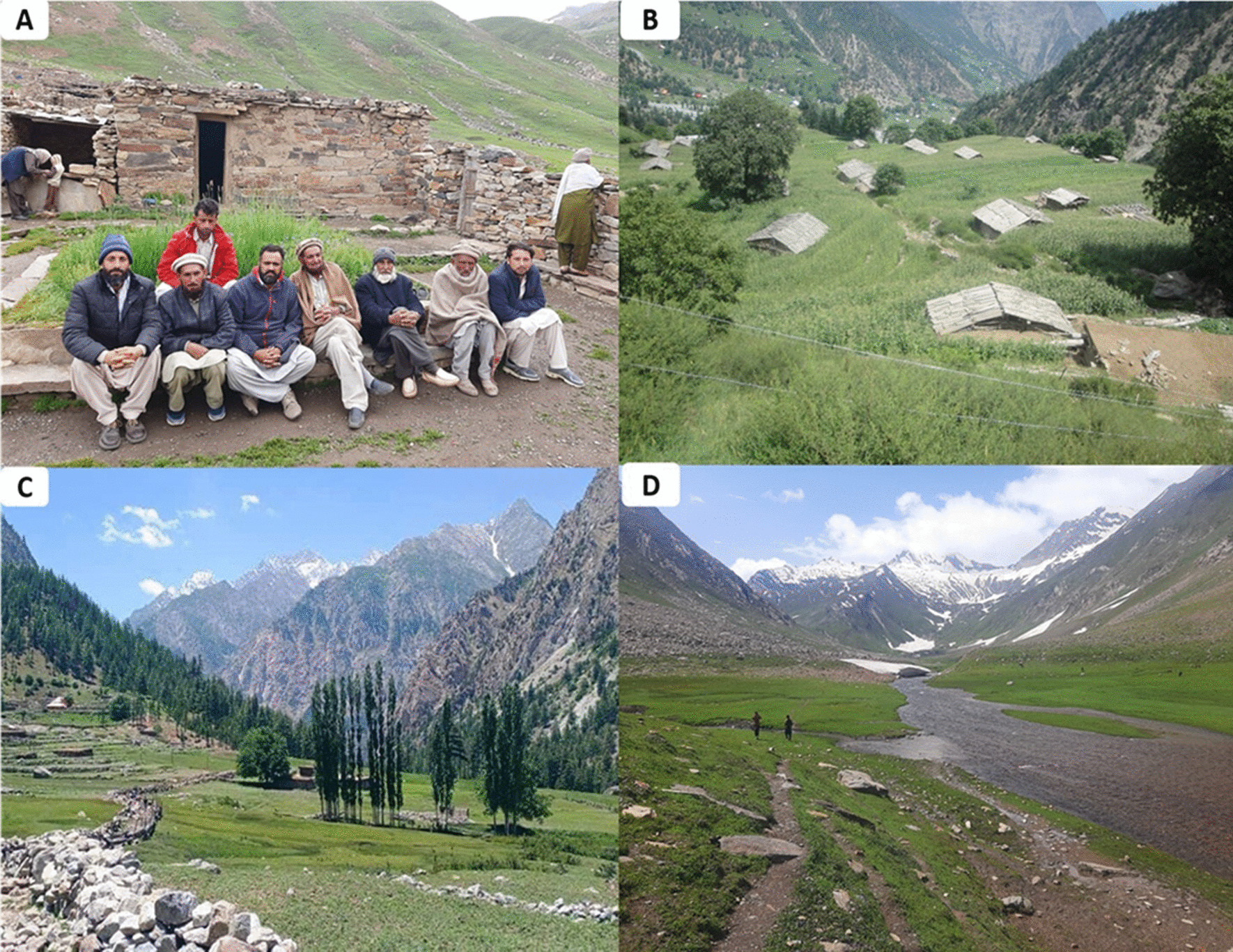
Panoramic view of study area. A. Authors with local informants in Supat valley, B. Razika village in Seo valley, C. Madian village in Kandian valley, D. Maheen village in Jalkot

Kohistan, which literally means the land of mountains, splits into three districts Kohistan Upper, Lower Kohistan, and Kolai-Palas Kohistan. The region lies between 35° 16′ 16″ N latitude, and 73° 13′ 24″ E longitude (Fig. 1), in the sub-humid eastern and wet mountain zones of north Pakistan. Precipitation in winter is in the form of rain in valleys, and snow and ice on the alpine pastures and hilly slopes [14]. The vegetation of Kohistan falls in dry temperate and moist temperate zones with dominant Himalayan moist temperate forests [15, 16]. The Indus River after crossing Gilgit Baltistan enters Kohistan Upper and divides it into North and South parts containing ridges of HKH region [17]. Because of its mountains, pasture lands and valleys (Fig. 2), Kohistan Upper is rich in diverse types of plants and animal species. Being the most remote area of Pakistan, Kohistan Upper lacks modern facilities. Therefore, local communities are mostly marginalized and depend on flora and fauna of their surroundings to meet food, medicines, fodder, fuel, and shelter requirements. These communities possess significant traditional knowledge on plant resources of the area. Local inhabitants of Kohistan Upper possess significant traditional knowledge (TK), and use plant resources as food, in primary health care systems, and for livelihoods in daily life. However, like other parts of the world, the young generation of Kohistan Upper is also under the influence of modernization. Specifically, recent developments under the China Pakistan Economic Corridor (CPEC) project are one of the key factors diverting the attitude of the young generation, mainly in Kohistan Upper. Therefore, it is essential to document the bio-culture heritage of this region.

Total population of Kohistan Upper is 306,337 [18]. The literacy rate of Kohistan Upper is 10.3% (male = 17.11%, female = 2.95%). All Kohistani are Sunni Muslims, and they accepted Islam in eighteenth century [19]. However, according to the local inhabitants, conversion of Kohistani to Islam is due to influence of two Pakhtun Saints namely Akhund Darwaza and Chishti saint Pir Baba of Parcha in Buner in sixteenth and seventeenth centuries. According to the historical record, the Kohistani community inhabited the region of Kohistan between the third and seventeenth centuries [20]. However, no exact period of their arrival has been confirmed yet from any historical documents [19]. Kohistani are mostly endogamic, and the tribes living on both sides of the Indus River are divided into various subtribes and castes. They have a unique socio-cultural structure that is rooted in strong traditions and customs. Each subtribe or caste except marginalized communities have one tribe leader called Malak. The Malak is an authoritative person who takes decisions on behalf of the tribe council (Jargah). Kohistani people consider themselves entirely different from other communities of the northern part of Pakistan and believe that they have different ancestry. The Kohistan is an area of male-dominated, and polygamous society. The marriage system in Kohistan is different from the rest of Pakistan. In the wedding ceremony, the bride's wedding dress is specially designed with local art, while the groom has no special dress code for the marriage ceremony [21].

Kohistani live entirely in the rural areas. Seo, Jalkot and Kandia are three main valleys of Kohistan Upper. Each valley has a specific language, culture, and historic background. KCs of these valleys are divided into three linguistic groups: Kohistani, Shina, and Gujjari. The Kohistani language is also known as Shuthun, Maiya, Indus Kohistani and Abasin Kohistani [22]. Kohistani is the language of the local community named Kohiste. They are 44.5% of the total population and live in Seo and Kandia valleys on the west bank of River Indus in Hindu Kush Mountain range. Most of these valleys speak Kohistani, except in the far-flung areas where Gujjar tribes speak Gujjari language. The Shinaki community of Jalkot located in the Himalayan Mountain range speaks Shina. They represent 45% of the total population and live on the east side of the Indus River. The nomadic Gujjar tribes live on both sides of the Indus River in pasture lands, and at scattered pits in the deep valleys of Seo, Jalkot and Kandia. It has been reported that Gujjars came to this region from Mongolia during Mongol incursion on India in the fifth century [23]. These tribes speak Gujjari, which accounts for 10.5% compared to other Kohistani and Shina [24, 25].

### Data collection

Field surveys were conducted from April 2020 to September 2021, and information was gathered from different linguistic groups viz. Kohistani, Shinaki and Gujjari. As illustrated in Fig. 1, these communities were dispersed in 12 different villages of Kohistan Upper. Four sites of each linguistic group were targeted during the survey including > 35 informants from each valley (Table 1).

**Table 1:** Characteristics of the targeted localities and study participants

Sites	Forest types	Ethnic group	AH	SA	EnR./ExR	Linguistic group	Village	El. (m,a,s,l.)	NH	NI (M/F)	AG (years)
Seo valley	Himalayan dry and moist temperate forests	Seowos (96%)	Sixteenth—seventeenth century	Pastoralism, animal husbandry and farming	Endogamic, rarely exogamic	Kohistani	Seo	878	320	13/5	15–60
							Razika	1830	275	15/3	
							Chaprow	1316	35	10/4	
		Gujjars/Guzar (4%)	NR	Pastoralism and animal husbandry	Exogamic	Gujjari	Zalii	3093	37	12/3	
Jalkot valley	Himalayan dry and moist temperate forest, and alpine pastures	Shinaki/Jalkoti (90%)	Sixteenth—seventeenth century	Pastoralism, animal husbandry, farming, and mining	Endogamic, rarely exogamic	Shina	Maheen	3503	25	15/0	20–80
							Sasak	2001	120	13/1	
							Sazen	1415	154	14/0	
							Jalkot	892	250	15/2	
		Gujjars/Guzar (10%)	NR	Pastoralism and animal husbandry	Exogamic	Gujjari	Gotay Baig	4741	25	9/0	
Kandia valley	Himalayan dry and moist temperate forests	Kheloos (85%)	Sixteenth—seventeenth century	Pastoralism, animal husbandry and farming	Endogamic, rarely exogamic	Kohistani	Khowr	1633	75	11/1	20–80
							Gabriyal	2113	286	12/2	
		Gujar/Guzar (15%)	NR	Pastoralism and animal husbandry	Exogamic	Gujjari	Maidan	2655	85	11/0	

AH, Arrival history in the area; SA, Subsistence activities; EnR, Endogamic rules; ExR, Exogamic rules; El, Elevation; NH. Number of households, NI, Number of informants; M, Male; F, Female; AG, Age groups; NR, No record

**Fig. 3:**
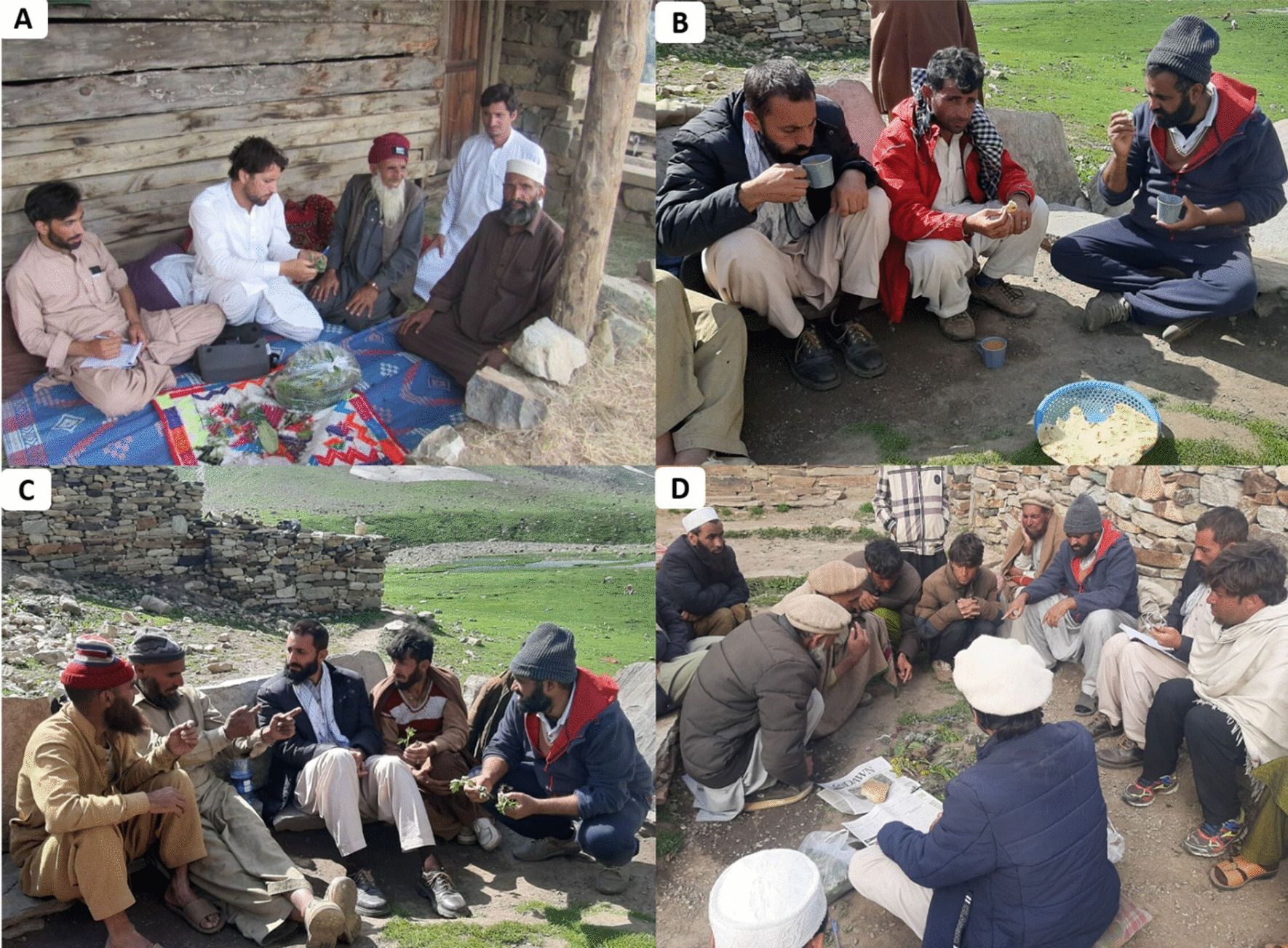
Data collection local informants. A. Interview with Kohistani linguistic group in Seo valley, B. Authors in field eating local food in Maheen village. C. Discussion with Gujari linguistic group in Supat valley, D. Authors taking information from Shina linguistic group at Jalkot valley

During the field survey, the guidelines endorsed by the International Society of Ethnobiology [26] were followed. Before the interviews, the objectives of the study were shared with local informants and then, informed verbal consent was taken for the collection and sharing of their knowledge/information with the public as reported earlier [8]. In addition, verbal permission was also received from local participants for the publication of field photographs (Fig. 3) taken during the survey. All participants aged between 15 and 80 years were selected from different groups based on the snowball technique [8]. From each linguistic group > 35 informants including aged/wise male and female, local herbalists and shepherds were the targeted participants. Data were collected from 171 informants including 88% male and 12% female (Table 1), using semi-structured interviews and field walks. Interviews and discussions on wild food plant species were carried out in local linguistics, i.e., Kohistani, Shina, and Gujjari. After the interview with the local informants, a group discussion was conducted to document more information regarding traditional uses of wild flora.

Afterward, all information was translated into English. Interviews and group discussions were mainly focused on the collection of wild food plant species (WFPs), and their mode of consumption as cooked vegetables, raw fruits, and salad, as flavoring agents in cooked food and for making tea. The local name of each plant, part(s) used, collection site(s), harvesting season/time, mode of preparation, and uses were documented. In addition, specific questions on animal-based dairy products prepared by local communities using traditional methods were also asked from local informants.

### Plant specimen collection and processing

All quoted WFPs, i.e., herbs, shrubs, and trees were collected along with field photographs from the fields with the help of local informants. Gathered specimens were identified with the help of taxonomic databases of Pakistan, i.e., ‘Flora of Pakistan’ [27–30], and by expert taxonomists. Botanical names and families of the identified specimen were further confirmed using international plant databases: The World Flora Online [31] and the Angiosperm Phylogeny website [32]. All specimens were properly dried, poisoned, mounted on herbarium sheets, provided with voucher numbers and deposited in the herbarium at COMSATS University Islamabad, Abbottabad Campus for future record.

### Use category and use report

Field data on WFPs were categorized into various groups based on distinct categories, i.e., vegetables, fruits, flavoring agents, salad, and tea, etc. Afterward, use report (UR), which is one of the most important tools used to calculate the cultural importance of plant species, was calculated following the method as explained by Tardío and Pardo-de-Santayana [33]. Use report was intended with the help of formula as mentioned below:

$$\mathrm{URs}={\sum }_{u=ui}^{uNC} {\sum }_{i=i1}^{iN}\mathrm{ UR}ui$$

URs were calculated by adding use report by all informant from *i* = 1 to *i* = *N* in different use category for WFPs. First, we sum the number of informants who reported each use category for WFPs, in the next step UR of each use category from u1 to uNC was added.

### Data analysis

Free software available at (http://bioinformatics.psb.ugent.be/webtools/Venn/ [05 January 2022]) was used to draw proportional Venn diagrams for the comparative assessment of reported WFPs among different linguistic groups. Final data were presented in tabular and graphical formats using MS Excel and Sigma Plot v12. The present ethnobotanical data were compared with existing literature of Pakistan [4–9, 34–52], and neighboring countries [53–57] to identify the novel uses of the current study.

## Result and discussion

### Uses of WFPs

A total of 64 plant species belonging to 54 genera from 45 botanical families were documented (Table 2), which were quoted as WFPs by the studied linguistic groups. Polygonaceae was the most dominant family with 8 spp., followed by Rosaceae (6 spp.), and Amaranthaceae (3 spp.), while the rest of the families were represented by 2 or 1 species. Our findings are in line with previous reports [4, 9, 45, 58–60] that have also reported these families frequently. The Polygonaceae was represented by *Bistorta amplexicaulis*,* Oxyria digyna*,* Persicaria amplexicaulis*,* Rheum australe*,* R. emodi*,* Rumex acetosa*,* R. dentatus*,* R. hastatus*, and the higher number of taxa from this family could be referred to its wide ecological distribution in the mountain areas of this region [9, 45, 58, 59]. Most of the members of this family are found in the high mountain areas, so the local people collect them in the summer season from pastures when bringing their animals to the high mountains. It is important to note that the wild vegetables are particularly collected by women. Female informants mentioned that they usually prefer vegetables with sour taste, because such vegetables have good flavor and keep stomach healthier. Mostly, these vegetables are consumed after cooking in water or buttermilk. However, fresh leaves of *Oxyria digyna* and *Rumex* species are eaten raw as snacks.

**Table 2:** Recorded wild food plants and their local uses

S. no.	Scientific name/family Voucher number	Local name	Habit	Part used	UC	Preparation	UR	ULG			PRFP	PRFNR
								G	K	S		
1	*Ajuga integrifolia* Buch.-Ham. Lamiaceae CUHA-06	Bhuti^K,S,G^	Herb	Ap	Cooked	Fresh aerial parts are boiled in water and consumed along with butter or ghee	74	+	+	−	No	No
2	*Allium carolinianum* Redouté Amaryllidaceae CUHA-231	Jangli peyaz^K^, Jangli poloond^S^	Herb	Ap	Consumed raw	Fresh aerial parts eaten raw as salad	23	−	+	++	Yes [5, 8]	No
3	*Amaranthus hybridus* L. Amaranthaceae CUHA-07	Gannar^K^, Ganaara^S^, Ganaar^G^	Herb	Ap	Cooked	Fresh aerial parts are boiled in water or Buttermilk, then squeezed with hands. After that, fried in homemade butter or ghee along with onion and common salt	118	−	+	+	Yes [6]	No
4	*Amaranthus viridis* L*.* Amaranthaceae CUHA-08	Gha gha^K^	Herb	Ap	Cooked	Fresh aerial parts are boiled in water, then squeezed with hands. After that, fried in homemade butter or ghee along with onion and common salt	9	−	++	–	Yes [5, 45]	No
5	*Barbarea verna* (Mill.) Asch. Brassicaceae CUHA-244	Sunnar^G^	Herb	Ap	Cooked	Fresh aerial parts are boiled in water, then squeezed and fried in homemade butter or ghee along with onion and salt	9	++	–	–	No	No
6	*Bistorta amplexicaulis* D.Don. Ronse Decr. Polygonaceae CUHA-15	Roye^K^, Mashlond^G^	Herb	Lvs	Cooked	Fresh leaves boiled in water. Then fried in butter or ghee along with onion and salt	11	−	++	–	Yes [41]	No
7	*Cedrus deodara* (Roxb. ex D.Don) G.Don Pinaceae CUHA-17	Beech^K^, Poloz^P^, low^G^	Tree	Res	Consumed raw	Resin is collected from trunk of the tree in sold form and chewed as babul gum. The solid resin is stored for future and is also given to friends for chewing in the lower areas. It is useful for strengthen the mouth gums and teeth	47	+	+	−	Yes [52]	No
8	*Celtis caucasica* (Willd.*)* Cannabaceae CUHA-19	Meyoon^K,S^ Dadoo^G^	Tree	Fr	Consumed raw	Fresh fruit is eaten as raw	61	−	+	+	Yes [46]	No
9	*Celtis australis* L*.* Cannabaceae CUHA-18	Meyo^K^	Tree	Fr	Consumed raw	Fresh fruit is eaten as raw	18	−	++	–	Yes [6]	No
10	*Chenopodium album* L. Amaranthaceae CUHA-21	Kawa^K^, Pathow^G^	Herb	Lvs	Cooked	Fresh leaves are boiled in water, then fried in butter or ghee along with onion and common salt	62	−	++	–	Yes [35]	No
11	*Cirsium arvense* (L.) Scop. Asteraceae CUHA-23	Harul^K^, Horal^G^	Herb	Lvs	Cooked	Fresh leaves are boiled in water, then fried in butter or ghee along with onion and common salt	42	+	++	–	Yes [5]	No
12	*Citrus aurantium* L. Rutaceae CUHA-25	Narang^S^	Tree	Fr	Consumed raw	Fresh fruit is eaten as raw	23	−	++	+	Yes [49]	No
13	*Clematis grata* Wall. Ranunculaceae CUHA-26	Zulto^K^	Climber	Lvs	Cooked	Fresh leaves are boiled in water, then fried in butter or ghee along with onion and common salt	19	−	++	–	No	No
14	*Convolvulus arvensis* L. Convolvulaceae CUHA-27	Ero^K^, Kheroon^G^	Climber	Lvs	Cooked	Fresh leaves are boiled in water, then fried in butter or ghee along with onion and common salt	56	−	++	–	Yes [5]	No
15	*Diospyros lotus* L. Ebenaceae CUHA-32	Amblook ^K, S^, Amlook^G^	Tree	Fr	Consumed raw	Ripened fruits are eaten as raw. Fruits are also dried and used in winter season	98	−	+	+	Yes [38, 40]	No
16	*Duchesnea indica* (Andrews) Teschem. Rosaceae CUHA-263	Bangros^K^, Makbursa^S^, Dharti mian^G^	Herb	Fr	Consumed raw	Fresh fruit is eaten as raw	65	+	+	+	Yes [37]	No
17	*Elaeagnus umbellata* Thunb. Elaeagnaceae CUHA-34	Gohi^S^	Herb	Fr	Consumed raw	Fresh fruit is eaten as raw	30	−	–	+ +	No	Yes [53]
18	*Ficus palmata* Forssk*.* Moraceae CUHA-37	Phah^K^, Pagoye^S^, Camera^G^	Tree	Lvs Fr	Cooked Consumed raw	Fresh leaves are boiled in water, then fried in butter or ghee along with onion and common salt Ripened fruits are eaten as raw	91	−	+	+	Yes [45, 60]	No
19	*Hypericum perforatum* L. Hypericaceae CUHA-41	Esperke^K^	Herb	Ap Lvs	Cooked Tea	Fresh aerial parts are boiled in water, then fried in butter or ghee along with onion and common salt. Leaves are dried in shade and consume as a tea	13 3	−	++	–	Yes [35]	No
20	*Impatiens edgeworthii* Hook. f. Balsaminaceae CUHA-42	Khanroye^K^, Benthal^G^	Herb	Sed Lvs	Consumed raw Cooked	Seeds are eaten as raw Fresh leaves are boiled in water, then fried in butter or ghee along with onion and common salt	20 9	+	++	–	No	No
21	*Indigofera tinctoria* L. Leguminosae CUHA-43	Kayth^K^, Kasti^S^, Kheenthey^G^	Shrub	Fl	Consumed raw	Fresh flowers are eaten as a raw	58	−	++	−	No	No
22	*Juglans regia* L. Juglandaceae CUHA-45	Choo^K^, Achoye^S^, Akhory^G^	Tree	Fr	Consumed raw	Fresh fruit kernel is eaten as a raw. Also dried, stored, and eaten in winter as dry fruit and mixed in traditional desserts	81	−	+	−	Yes [6]	No
23	*Juniperus excelsa* M.Bieb. Cupressaceae CUHA-279	Cheli^S^	Tree	Bk	Ash	Bark is burnt to make ash, and this as is used in snuff as flavoring agent	9	−	–	+ +	No	No
24	*Leontopodium himalayanum* DC. Compositae CUHA-280	Taliban ka qaleel^S^	Herb	Ap	Consumed raw	Aerial parts are chewed as babul gum	11	−	–	+ +	No	No
25	*Malus sylvestris* (L.) Mill. Rosaceae CUHA-285	Bhap^K^, Paloye^S^, Bhabuyee^G^	Tree	Fr	Consumed raw	Fresh fruits are eaten as raw, also dried, and stored for winter season	64	+	+	+	Yes [38]	No
26	*Mentha longifolia* (L.) L. Lamiaceae CUHA-48	Phemel^K^, Phebel^S^, Podina^G^	Herb	Ap Lvs	Cooked Consumed raw Tea	Fresh aerial parts are boiled in water, then fried in butter or ghee along with onion and common salt Fresh leaves are used as salad Fresh and dried leaves are boiled in water and used as tea	106 10 9	−	+	+	Yes [36]	No
27	*Morus nigra* L. Moraceae CUHA-49	Marash^K^, Marosh^S^, Korow^G^	Tree	Fr	Consume raw	Fresh fruits are eaten as raw. Fruits are also dried and stored for use in winter season	81	−	+	+	Yes [7]	No
28	*Myrtus communis* L*.* Myrtaceae CUHA-291	Ambo^K^, Aob^S^	Shrub	Lvs Fr	Tea Consumed raw	Fresh and dried leaves are boiled in water to make tea Fresh fruits are eaten raw	11 43	−	+	+ +	Yes [35]	No
29	*Nasturtium officinale* R.Br. Brassicaceae CUHA-50	Talib shah^K^	Herb	Ap	Cooked	Fresh aerial parts are boiled in Buttermilk. After that fried in homemade butter or ghee along with onion and common salt	23	−	++	–	Yes [35]	No
30	*Olea ferruginea* Wall. ex Aitch. Oleaceae CUHA-52	Kowo^K,S,G^	Tree	Fr	Consumed raw	Fresh fruits are eaten as raw	27	+	+	+	Yes [52]	No
31	*Oxalis corniculata* L. Oxalidaceae CUHA-53	Cheko daro^K^, Chiki rang^S^, Terko^G^	Herb	Ap Lvs	Cooked Consumed raw	Fresh aerial parts are boiled in Buttermilk. Then fried in homemade butter or ghee along with onion and salt Fresh leaves are used as salad	82 17	+	+	+	Yes [6]	No
32	*Oxyria digyna* Hill Polygonaceae CUHA-220	Huli^S^	Herb	Ap	Cooked	Aerial parts are boiled in water or Buttermilk. Then fried in butter or ghee along with onion and salt	13	−	–	+ +	No	Yes [56]
33	*Parrotiopsis jacquemontiana* Rehder Hamamelidaceae CUHA-294	Pashoo^K^, Pashoot^S^, Passat^G^	Shrub	Fr	Consumed raw	Fresh fruit are eaten as raw	30	+	+	+	No	No
34	*Pedicularis oederi* Vahl Orobanchaceae CUHA-296	Khana Phor^S^	Herb	Lvs	Cooked	Fresh leaves are boiled in water or Buttermilk along with other edible herbs. After that, fried in homemade butter or ghee along with onion and salt	7	−	–	+ +	No	Yes [57]
35	*Persicaria amplexicaulis* (D. Don) Ronse Decr. Polygonaceae CUHA-57	Roye^K^	Herb	Lvs	Cooked	Fresh leaves are boiled in water, then fried in butter or ghee along with onion and common salt	10	−	++	–	Yes [49]	No
36	*Pinus gerardiana* Wall*. ex* D.Don Pinaceae CUHA-61	Chonge^K^, Tholash^S^, Shentah^G^	Tree	Fr	Consumed raw	Fruits are eaten as raw, also dried, and used in winter	68	−	++	−	Yes [6]	No
37	*Pistacia khinjuk* Stocks Anacardiaceae CUHA-63	Khakow^K^	Tree	Fr	Consumed raw	Fruits are eaten as raw	13	−	++	–	Yes [52]	No
38	*Podophyllum hexandrum* Royle Berberidaceae CUHA-66	Shargoye^K^, Khakori^G^	Herb	Fr	Consumed raw	Fruits are eaten as raw	35	++	+	–	Yes [51]	No
39	*Portulaca oleracea* L. Portulacaceae CUHA-72	Pechel^K,G^	Herb	Ap	Cooked	Aerial parts are boiled in water or Buttermilk. After that fried in homemade butter or ghee along with onion and salt. Aerial parts are also half boiled in Buttermilk, then squeezed, dried, and stored for winter season	27	++	–	–	Yes [5]	No
40	*Primula elliptica* Royle Primulaceae CUHA-302	Moril^S^	Herb	Ap	Flavoring agent	Aerial parts are dried, and grinded to make powder, which is used in snuff as a flavoring agent	11	−	–	+ +	No	No
41	*Primula macrophylla* D. Don Primulaceae CUHA-303	Kamzoor Moril^S^	Herb	Ap	Flavoring agent	Aerial parts are dried, and grinded to make powder, which is used in snuff as a flavoring agent	9	−	–	+ +	No	No
42	*Prunus armeniaca* L*.* Rosaceae CUHA-73	Jarot^S^, Hari^G^	Tree	Fr	Consumed raw	Fresh fruits are eaten as raw. Fruits are also dried in sun light, then stored for use in winter season	32	−	–	+ +	Yes [38]	No
43	*Prunus persica* (L.) Batsch Rosaceae CUHA-74	Aro^K^, Aar^S^, Aro^G^	Tree	Fr	Consumed raw	Fresh fruits are eaten as raw	44	−	++	−	Yes [52]	No
44	*Punica granatum* L*.* Lythraceae CUHA-78	Dango^K^, Daroye^S^, Daroo^G^	Tree	Fr	Consumed raw	Fresh fruits are eaten as raw	54	−	-	+	Yes [37]	No
45	*Quercus semecarpifolia* Sm*.* Fagaceae CUHA-81	Ban^K^, Bani^S^, Terry^G^	Tree	Fr	Cooked	Fresh fruits are roasted and eaten as raw	104	−	+	+	Yes [44]	No
46	*Ranunculus laetus* Wall. ex Hook.f. & Thomson Ranunculaceae CUHA-82	Marchake^K^, Marchakaye^G^	Herb	Ap	Cooked	Aerial parts are boiled in water. Then fried in homemade butter or ghee along with onion and salt	20	++	–	–	No	No
47	*Rheum australe* D. Don Polygonaceae CUHA-311	Chutiyal^K,S,G^	Herb	Stm	Consumed raw	Fresh stem is peeled, and inner part is eaten	32	+	+	+	Yes [63]	No
48	*Rheum emodi* Wall. Polygonaceae CUHA-312	Chutiyal^K,S,G^	Herb	Stm	Consumed raw	Fresh stem is peeled, and inner part is eaten	36	+	+	−	Yes [63]	No
49	*Rubus fruticosus* L. Rosaceae CUHA-88	Ancha^K^, Goracha^G^	Shrub	Fr	Consumed raw	Fresh fruits are eaten	65	−	++	–	Yes [43]	No
50	*Rubus niveus* Thunb. Rosaceae CUHA-89	Ancha^S^	Shrub	Fr	Consumed raw	Fresh fruits are eaten	34	−	–	+ +	Yes [38, 41]	No
51	*Rumex acetosa* L. Polygonaceae CUHA-89	Chuki^S^	Herb	Ap	Cooked	Aerial parts are boiled in water or Buttermilk along with other edible herbs. After that, fried in homemade butter or ghee along with onion and salt	10	−	–	+ +	No	Yes [54]
52	*Rumex abyssinicus* Jacq*.* Polygonaceae CUHA-90	Markash^K^	Herb	Fr Lvs	Consumed raw Cooked	Ripened fruits are eaten Aerial parts with other edible herbs are boiled in water or Buttermilk. Then fried in butter or ghee along with onion and salt	12 10	−	++	–	No	Yes [55]
53	*Rumex dentatus* L. Polygonaceae CUHA-91	Babal^K^, Hububal^S^, Hulow^G^	Herb	Lvs	Cooked	Aerial parts along with other edible herbs are boiled in water or Buttermilk. After that fried in homemade butter or ghee along with onion and salt	81	+	+	−	Yes [8, 45]	No
54	*Rumex hastatus* D. Don Polygonaceae CUHA-92	Chekow^S^, Huli^G^	Herb	Ap Lvs	Cooked Consumed raw	Aerial parts are boiled in water or Buttermilk. Then fried in butter or ghee along with onion and salt Fresh leaves are eaten as salad	34 10	+	–	+ +	Yes [44]	No
55	*Solanum villosum* Mill. Solanaceae CUHA-330	Kuchmacho^K^, Kachmach^S^, Kuchmacho^G^	Herb	Lvs Fr	Cooked Consumed raw	Fresh leaves are boiled in water, then fried in butter or ghee along with onion and salt Fresh fruits are eaten as raw	90 11	−	+	−	Yes [50]	No
56	*Solanum nigrum* Acerbi ex Dunal Solanaceae CUHA-97	Ker ker^S^	Herb	Fr	Consumed raw	Fresh fruits are eaten	35	−	–	+ +	Yes [41]	No
57	*Taxus wallichiana* Zucc. Taxaceae CUHA-102	Thoon^K^	Tree	Fr	Consumed raw	Fresh fruits are eaten	12	−	++	–	Yes [41]	No
58	*Torilis leptophylla* Rchb.f. Apiaceae CUHA-341	Dhaniyal^S,G^	Herb	Ap	Cooked	Fresh aerial parts are boiled in water. After that fried in homemade butter or ghee along with onion and common salt	25	++	–	+	Yes [45]	No
59	*Trifolium pratense* L. Leguminosae CUHA-103	Kana eshpet^K^, Shotal^S^, Shotal^G^	Herb	Lvs	Cooked	Fresh leaves are boiled in water, then fried in butter or ghee along with onion and salt	41	+	−	−	Yes [45]	No
60	*Urtica dioica* L. Urticaceae CUHA-104	Zahoye^K^, Jam jama^S^, Keyori^G^	Herb	Ap	Cooked	Aerial parts are boiled in water along with other edible herbs. The fried in homemade butter or ghee along with onion and salt	90	+	+	−	Yes [38]	No
61	*Viburnum cotinifolium* D. Don Adoxaceae CUHA-107	Aoza^K^	Herb	Fr	Consumed raw	Fresh fruits are eaten	10	−	++	–	Yes [38]	No
62	*Viola pilosa* Blume Violaceae CUHA-108	Tekro^K^, Lilo^S^, Ratkari^G^	Herb	Ap	Cooked	Aerial parts are boiled in water along with other edible herbs. The fried in homemade butter or ghee along with onion and salt	86	+	+	+	No	Yes [47]
63	*Vitis jacquemontii* Roem. & Schult. Vitaceae CUHA-110	Jacha^K^, Zacha^S^, Daakh^G^	Climber	Fr	Consumes raw	Fresh fruits are eaten	89	+	+	+	Yes [42]	No
64	*Ziziphus jujuba* Mill. Rhamnaceae CUHA-112	Sagee^K^, Zazen^S^, Sageen^G^	Tree	Fr	Consumes raw	Fresh fruits are eaten. Also dried and stored for winter season	30	+	+	+	Yes [8]	No

K, Kohistani; S, Shina; G, Gujari; Ap, Aerial parts; Fr, Fruits; Lvs, Leaves; Stm, Stem; Sed, Seed; Fl, Flower; Res, Resin; Bk, Bark; UC, Use category; UR, Use report; ULG, Use in linguistic groups; – not reported; − less than 30% use reports; + less than 50% & more than 30% use reports; ++ more than 50% use reports; PRFP, Previous report as food from Pakistan; PRFNR, Previous report as food from neighboring regions

Rosaceae was the second dominant family (6 species), and the use of plant species belonging to this family is mainly attributed to the sweet taste of their fruits, which are consumed raw and are easily available in the maize belt and the lower area of Kohistan Upper. Similarly, the members of Amaranthaceae (3 species) are very frequently used as cooked vegetables as they are found mostly nearby homes and in agricultural lands as weeds. Other important botanical families are Lamiaceae, Brassicaceae, and Moraceae. The aromatic properties of Lamiaceae make the family favorite among many local communities across the globe [61, 62].

**Fig. 4:**
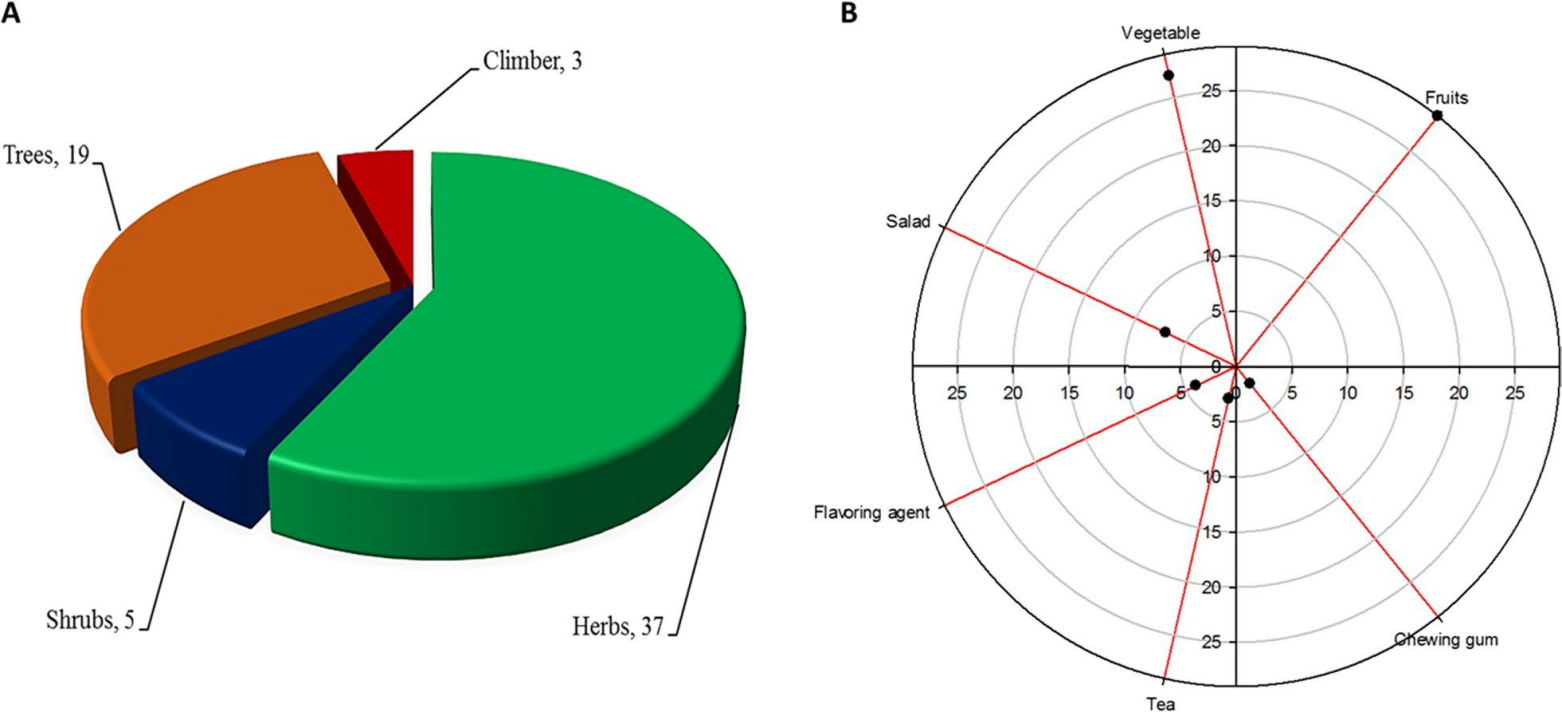
A. Different life forms of reported edible wild plant species, B. Use categories of WFPs

Among the reported plant species (Fig. 4A), 37 were herbs, followed by trees, shrubs, and climbers (19, 5, and 3 species in each life form). Both men and women collect the WFPs. Reported botanical taxa consumed as WFPs were mostly herbs which are usually harvested at their younger stages. Many participants mentioned that they collect leaves of *Amaranthus hybridus*,* A. viridis*,* Barbarea verna*,* Chenopodium album*,* Cirsium arvense*,* Ficus palmata*,* Rheum australe*,* R. emodi*,* Rumex dentatus* and *Urtica dioica* at their young stages. Particularly *U. dioica* and *R. dentatus* are gathered soon after the snow is melted when they are younger as in later stages it is difficult to pick the leaves of *U. dioica*, and the taste also becomes unpleasant. The herbaceous life form of the reported plants is preferred, and this might be due to the fact that the majority of the populace has the opportunity to collect herbs easily. Certain plant ingredients were collected and preserved for winters such as *Cedrus deodara*,* Diospyros lotus*,* Mentha longifolia*,* Morus nigra*,* Pinus gerardiana*,* Portulaca oleracea*,* Prunus armeniaca*,* Prunus persica*,* Punica granatum*,* Urtica dioica*,* Viola pilosa*, and *Ziziphus jujuba*.

Local inhabitants of the study area consume WFPs in different ways (Fig. 4B). Most of the reported taxa are consumed as fresh and dry fruits (29, 45.3%), cooked as vegetables (27, 42.1%), consumed as a fresh salad (7, 10.9%), used as flavoring agent (4, 6.2%), and in making tea (3, 4.6%). The resin and mucilaginous material from *Cedrus deodara* and *Leontopodium himalayanum* are used as chewing gum (2, 3.1%). Among the plant parts used (Table 2), local inhabitants consume fruits of 29 plant species, followed by aerial parts, leaves and stem (20, 19, and 2 species, respectively) as food. Whereas flowers, resin, and seeds of one species each are used as food.

**Fig. 5:**
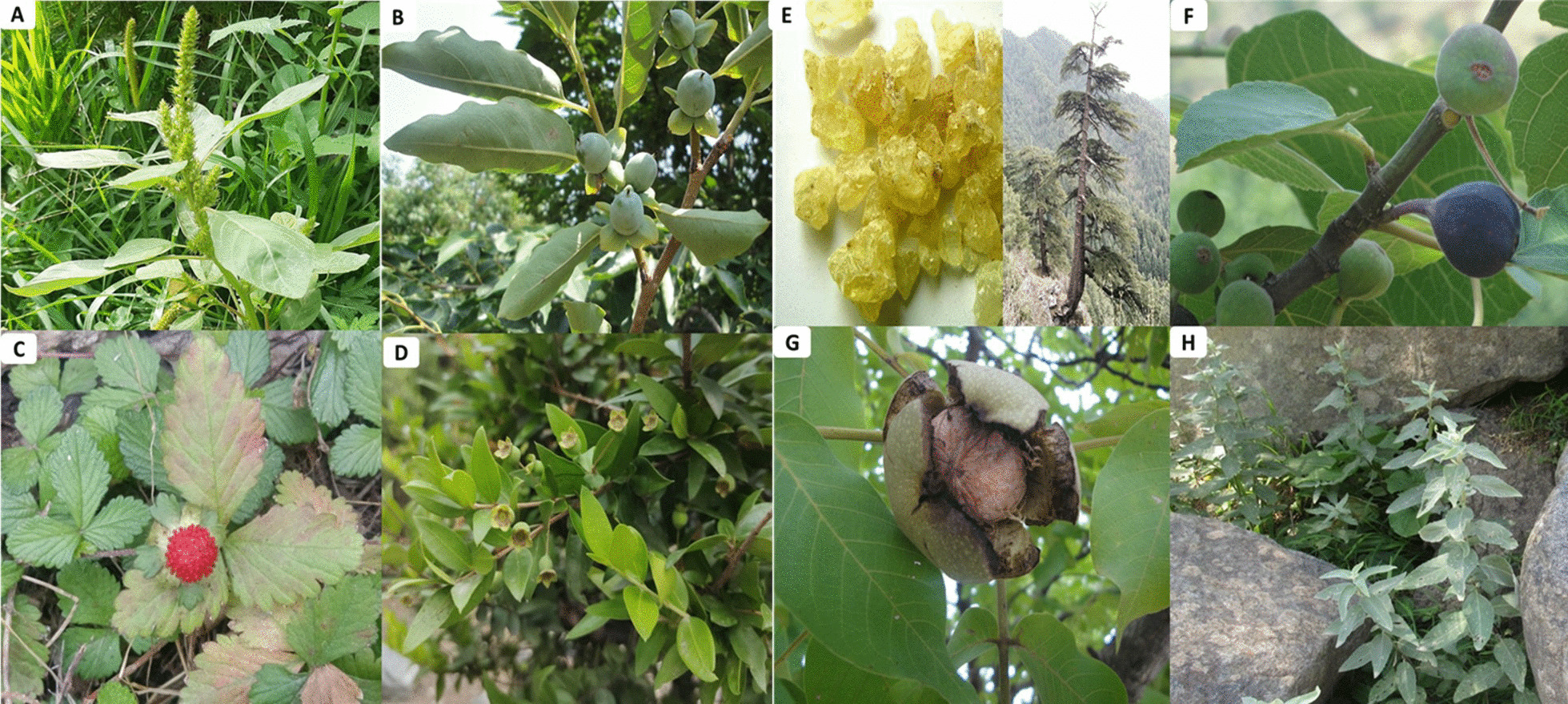
Most cited edible wild plant species of Kohistan Upper. **A** *Amaranthus hybridus*, **B** *Diospyros lotus*, **C** *Duchesnea indica*, **D** *Myrtus communis*, **E*** Cedrus deodara*, **F** *Ficus palmata*, **G** *Juglans regia*, **H** *Mentha longifolia*

Use reports (URs) of WFPs in different use categories are given in Additional file 1: Table S1. In total, 2853 URs were documented. Maximum URs were of fruits (1420 for 29 plants species), followed by vegetables (1036 URs/27 species), salad (205 URs/7 species), flavoring agent (111 URs/4 species), chewing gum (58 URs/2 species), and tea (23 URs/3 species). Some plant species that were frequently reported by the study participants are also shown in Fig. 5A–H. As mentioned in Table 2, among the reported taxa, *Quercus semecarpifolia* (104 URs),* Solanum villosum* (101 URs), *Diospyros lotus* (98 URs),* Ficus palmata* (91 URs), and *Duchesnea indica* (65 URs) were the most frequently quoted wild fruits. Amongst the wild vegetables, *Amaranthus hybridus* (118 URs),* Mentha longifolia* (106 URs),* Urtica dioica* (90 URs),* Viola pilosa* (90 URs),* Rumex dentatus* (81 URs),* Ajuga integrifolia* (74 URs),* Chenopodium album* (62 URs) and *Convolvulus arvensis* (56 URs) were highly quoted.

Certain taxa that were quoted by the participants to be used as fresh salad with maximum use reports were *Indigofera tinctoria* (58 URs),* Rheum emodi* (36 URs),* R. austral* (32 URs),* Impatiens edgeworthii* (29 URs), and *Allium carolinianum* (23 URs)*. Oxalis corniculata*,* Primula elliptica*,* P. macrophylla* and *Juniperus excelsa* are used as flavoring agents with maximum use reports (82, 11, 9, and 9, respectively). *Myrtus communis* was the most cited taxon (11 URs) used in making recreational tea, and similarly, *M. longifolia* (9 URs) and *Hypericum perforatum* (3 URs) are also important plants used in teas. Most of the WFPs have already been reported from other parts of Pakistan for different food purposes [4, 8, 9, 34, 35, 45, 60, 63–65]. Some species were also used as food–medicines as most of the informants described those who live close to forests at higher altitudes that they prefer resin of *C. deodara*, which is commonly chewed as gum to make their teeth and mouth gums stronger.

### Cross-analysis of traditional knowledge among linguistic groups

**Fig. 6:**
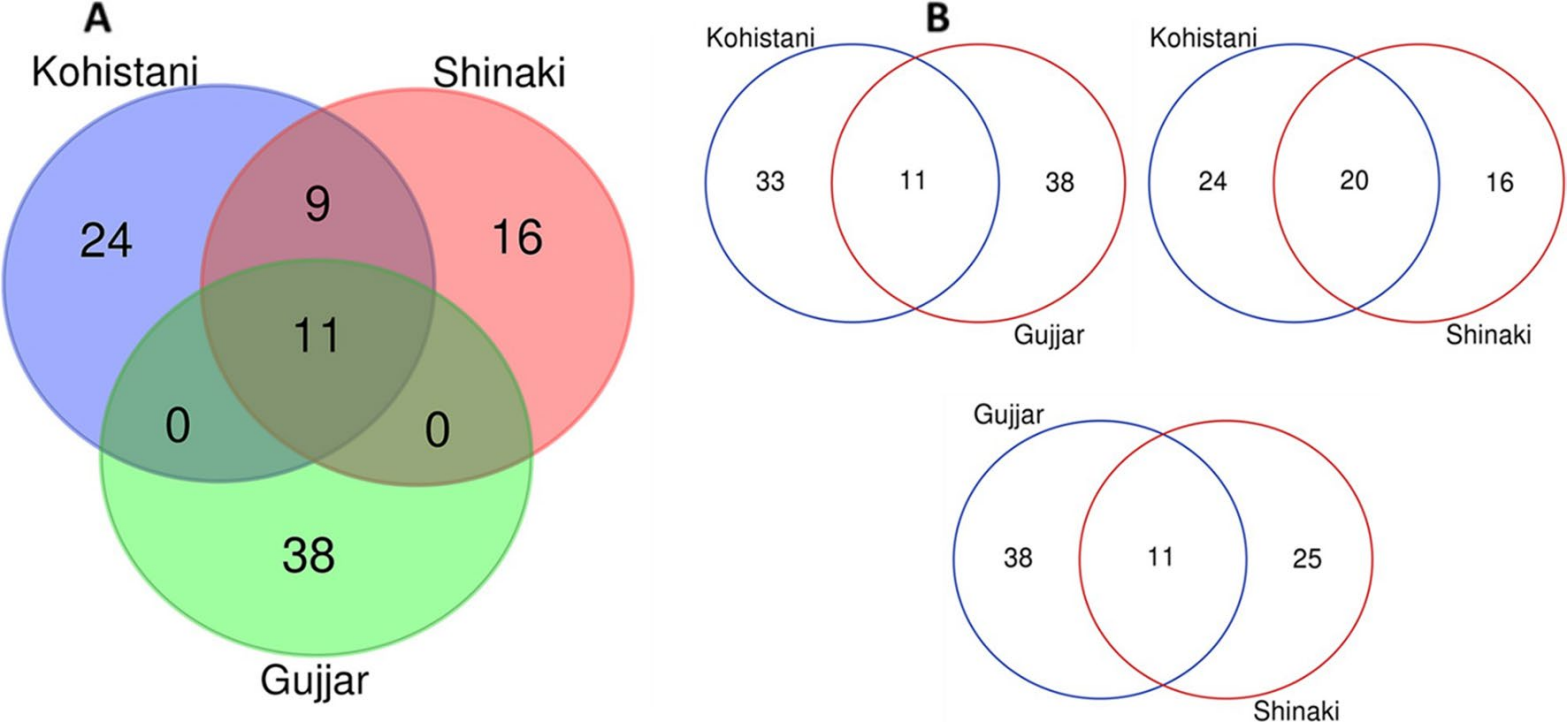
Venn diagrams showing cross-culture comparison of food uses of reported taxa among different linguistic groups of Kohistan Upper. **A** Indicates overall homogeneity and heterogeneity of WFPs among three linguistic groups, **B** shows homogeneity in uses of WFPs between linguistic groups

A cross-cultural comparison of the food ethnobotany of the three linguistic groups has revealed a high degree of heterogeneity, and only a small proportion (11 species, 14.1%) of food uses of certain recorded taxa were commonly shared among the three linguistic groups (Fig. 6A). In between three groups (Fig. 6B), maximum homogeneity was between Kohistani and Shinaki (20 species, 50.0%), followed by Gujjars and Shinaki (11 species, 17.4%), and Kohistani and Gujjars (11 species, 15.4%). Gujjars reside in the high-altitude valley of Kohistan Upper on both sides of the Indus River to live separately. However, they are compelled to do work for the Kohistani and Shinaki communities because they live in their lands and are mostly dependent on them. Therefore, the interaction of Gujjars with Kohistani and Shinaki is more than the interaction of Kohistani to Shina communities. Gujjars are grazing the cattle of Kohistani and Shinaki communities and look after the agricultural field and do labor work for them. Comparatively, the Gujjar community reported maximum uses (38, 48.7%), followed by Kohistani (24, 30.7%), and Shinaki (16, 20.5%). As mentioned in Table 2, Gujjars reported a smaller number of plants (40 species) compared to Kohistani and Shinaki (48 and 44 species, respectively), but they retained more traditional knowledge of their quoted plants in terms of use as compared to other two groups. This result might be because Gujjar people are living at higher elevations and they graze their animals at pastures, practice extensive transhumance pastoralism, and pass through different ecological landscapes thus having sufficient experiences with certain plants and retaining more knowledge. Although Gujjars retain more traditional knowledge on WFPs than other two linguistic groups (this could be due to the fact that in Kohistan they live in pastures at higher elevations where plant diversity is limited, specifically shrubs and trees are almost absent), they reported a smaller number of wild food plant species. In addition, they also prefer various dairy products, i.e., milk, butter, ghee, and buttermilk, etc., in their routine diet.

Some important plant species which were reported with idiosyncratic uses among Gujjars were *Barbarea verna*,* Duchesnea indica*,* Olea ferruginea*,* Oxalis corniculata*,* Parrotiopsis jacquemontiana*,* Portulaca oleracea*,* Punica granatum*,* Malus sylvestris*,* Ranunculus laetus*,* Rheum australe*,* Trifolium pratense*,* Viola pilosa*,* Vitis jacquemontii* and *Ziziphus jujuba*. It is important to know that transmigration has become one of the main components that helped to retain more local knowledge on diverse flora when they pass through different ecological landscapes. Such as Rana et al. [11] described that Gujjars have more traditional knowledge because of their nomadic lifestyle.

The remarkable heterogeneity in the use of WFPs among the three linguistic groups could be referred to as the lack of common practice of endogamy. For instance, Kohistani and Shinaki do not tend to intermarry with each other which may also have prevented the horizontal transmission of local knowledge [15]. Moreover, Gujjars tend to be exogamic toward Kohistani and Shinaki, but in return Kohistani and Shina do not prefer to intermarry with Gujjars.

All three groups live in separate valleys; therefore, geography has also played an important role in keeping the idiosyncrasy of the recorded food ingredients among the considered groups. However, only in the winter season at Komila Bazar (located west of the Indus River in Kohistani speaking belt) is the main place for their social interactions. It is important to note that language has also played an important role in shaping local ecological knowledge of WFPs. We also found some common local names among the three groups for certain taxa (Table 2), such as *Ajuga integrifolia* (Bhuti), *Olea ferruginea* (Kowo), *Rheum australe* (Chutiyal), and *Rheum emodi* (Chutiyal). This might be due to the fact that local communities are sharing knowledge on traditional uses of these species since long time with each other. Moreover, 34.3% WFPs were reported by all linguistic groups but with different name in Shinaki, Kohistani and Gujjari. Therefore, it is possible that these plant species have different routes of knowledge transformation. Rest of the species were reported by one or two communities with their local languages. The language of communication used in the main Bazar (Komila) is Kohistani language, which is the *lingua franca*. Currently, both linguistic (who- Shina and Kohistani) groups are residents in Komila Bazar, and the residents of these groups speak both languages but are limited to Komila Bazar. In summer villages (pastures) still have very limited interaction with each other.

Moreover, Kohistani community reported the maximum number of WFPs with unique uses compared to the Shinaki and Gujjars communities. The WFPs reported precisely by Kohistani community are *Celtis australis*,* Clematis grata*,* Convolvulus arvensis*,* Hypericum perforatum*,* Indigofera tinctoria*,* Nasturtium officinale*,* Persicaria amplexicaulis*,* Pistacia khinjuk*,* Rumex abyssinicus*,* Taxus wallichiana and Viburnum cotinifolium*. The WFPs unique to Shinaki community are *Elaeagnus umbellata*,* Juniperus excelsa*,* Leontopodium himalayanum*,* Oxyria digyna*,* Pedicularis oederi*,* Primula elliptica*,* Primula macrophylla*,* Rumex acetosa* and *Solanum nigrum*. Whereas WFPs which are explicitly preferred by Gujjars are *Barbarea verna*,* Portulaca oleracea*,* Ranunculus laetus*, and *Trifolium pratense*.

We have also found some important idiosyncratic uses for commonly shared taxa among the considered groups. For instance, Gujjars cooked young leaves of *Ficus palmata* in water and used them as vegetables, while Kohistani and Shinaki eat its ripened fruits only. Similarly, Gujjars cooked aerial parts of *Hypericum perforatum* as a vegetable, while Kohistani used its dried leaves as tea. Fresh leaves of *Mentha longifolia* are used as a flavoring agent in vegetables and meat by Gujjar people, but Kohistani uses these leaves as salad, while Shina speakers use them in making tea. Kohistani uses the leaves of *Myrtus communis* as a flavoring agent, and Shinaki uses these leaves as tea. Likewise, leaves of *Oxalis corniculata* are used as vegetables by Gujjars, but the Shina community used fresh leaves of this plant as a salad. Similar trends were noted for *Rumex hastatus* and *Solanum villosum.* These findings confirm that within the same area, different ethnolinguistic groups have diverse uses of plant species, and these variations may also be within the use of different parts of the same plant species [66].

### Comparison of present uses with reported literature

**Fig. 7:**
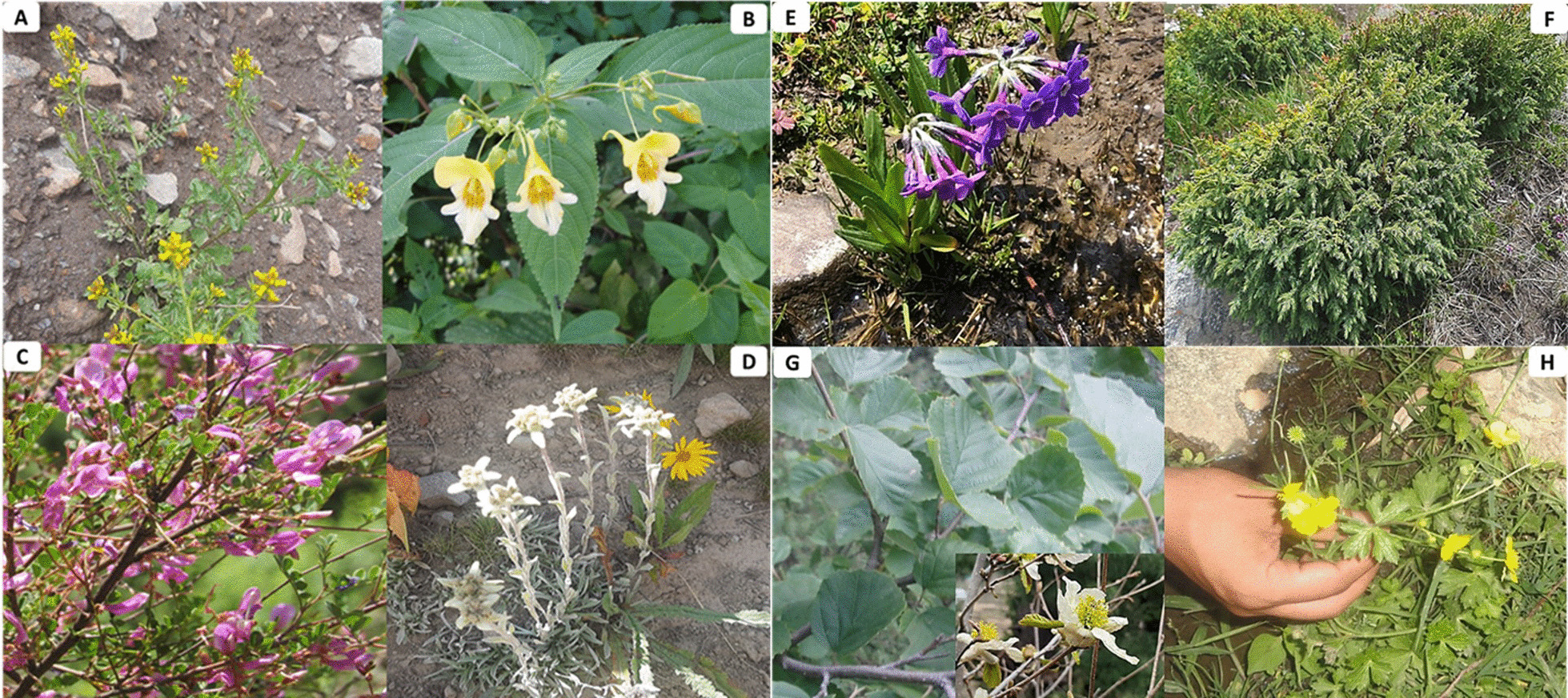
Newly reported edible wild plant species of Kohistan Upper. **A** *Barbarea verna*, **B** *Impatiens edgeworthii*, **C** *Indigofera tinctoria*, **D** *Leontopodium himalayanum*, **E** *Primula macrophylla*, **F** *Juniperus excelsa*, **G** *Parrotiopsis jacquemontiana*, **H** *Ranunculus laetus*

Out of 64 reported WFPs, (39, 60.9%) taxa were reported previously from different parts of Pakistan as wild food plants [4–8, 34, 35, 40, 41, 45, 63–65], and (20, 31.2%) were reported for the first time from the country, while (5, 7.8%) have already been reported from neighboring regions, i.e., India, China, Nepal, Turkey, and Bangladesh (Table 2). Some food ingredients were new to food ethnobotanical literature of Pakistan and were extracted from *Ajuga integrifolia*,* Barbarea verna*,* Clematis grata*,* Impatiens edgeworthii*, *Indigofera tinctoria*, *Juniperus excelsa*,* Leontopodium himalayanum*, *Parrotiopsis jacquemontiana*, *Primula elliptica*,* P. macrophylla*, and *Ranunculus laetus* (Fig. 7A–H). Although, *Clematis grata*,* Elaeagnus umbellata*, and *Trifolium pratense* have been reported as medicinal plants from different areas of Pakistan [36–40, 45, 58, 67], but were recorded as WFPs in the current study. Likewise, *Cedrus deodara* has been reported as timber wood [7], but inhabitants of Kohistan Upper also use its resin as chewing gum. Fruit of *Ficus palmata* is edible and eaten raw when ripened [35, 41], but in Kohistan Upper its young leaves are cooked in Buttermilk as vegetable. Wood of *Parrotiopsis jacquemontia* has been used in making agricultural tools [34, 52], but local inhabitants of Kohistan Upper consume its fresh fruit orally. It was observed that *Oxyria digyna*,* Rumex acetosa*, *Rumex abyssinicus*, *Viola pilosa* are used as WFPs only in Kohistan Upper but have never been reported from other parts of Pakistan. However, these species have also been reported as wild foods from Turkey, Bangladesh, and India [47, 54–56]. This might be due to the abundance of these plant species in the study area, marginalization, poverty, and pastoralism, which are the key factors in shaping the human–environment relationship [68, 69]. However, the maximum number of use reports for *Q. semecarpifolia*,* S. villosum* as fruit,* A. integrifolia*,* U. dioica*, and *V. pilosa* as vegetable, *I. tinctoria*,* R. emodi*,* R. australe*, and *I. edgeworthii* as salad,* M. communis* and *H. perforatum* as tea, *C. deodara* and *L. himalayanum* as chewing gum were rarely reported before.

### Traditional gastronomy and food tourism

Foraging of WFPs is an important ecological practice among the local communities. This act is also coupled by horticularism and extensive pastoralism to obtain ingredients for their local food systems and support local subsistence economies. Local communities of Kohistan Upper keep many goats, sheep, cows, and buffalos. Some of the important crops including local varieties of maize (yellow and white), and wheat are grown in the area. It is important to mention that their local food system is mainly composed of dairy products obtained from their domestic animals. Thus, along with foraging of WFPs, people also grow tomato, onion, potato, soybean, radish, Amaranthus and lady’s finger in their home gardens. We have recorded some important food ingredients which were quite famous and were used by the local communities in different seasons. For instance, *Portulaca oleracea*, *Mentha longifolia*, and *Amaranthus hybridus* are not only consumed fresh, but they are also dried and preserved for further use in winter. Before preservation, fresh plant materials are half cooked in water, squeezed and dried in sunlight, then they are stored in cotton sacks for the winter season. Some fruits such as *Diospyros lotus*,* Z. jujuba*, and *Juglans regia* are dried and used in winter.

**Fig. 8:**
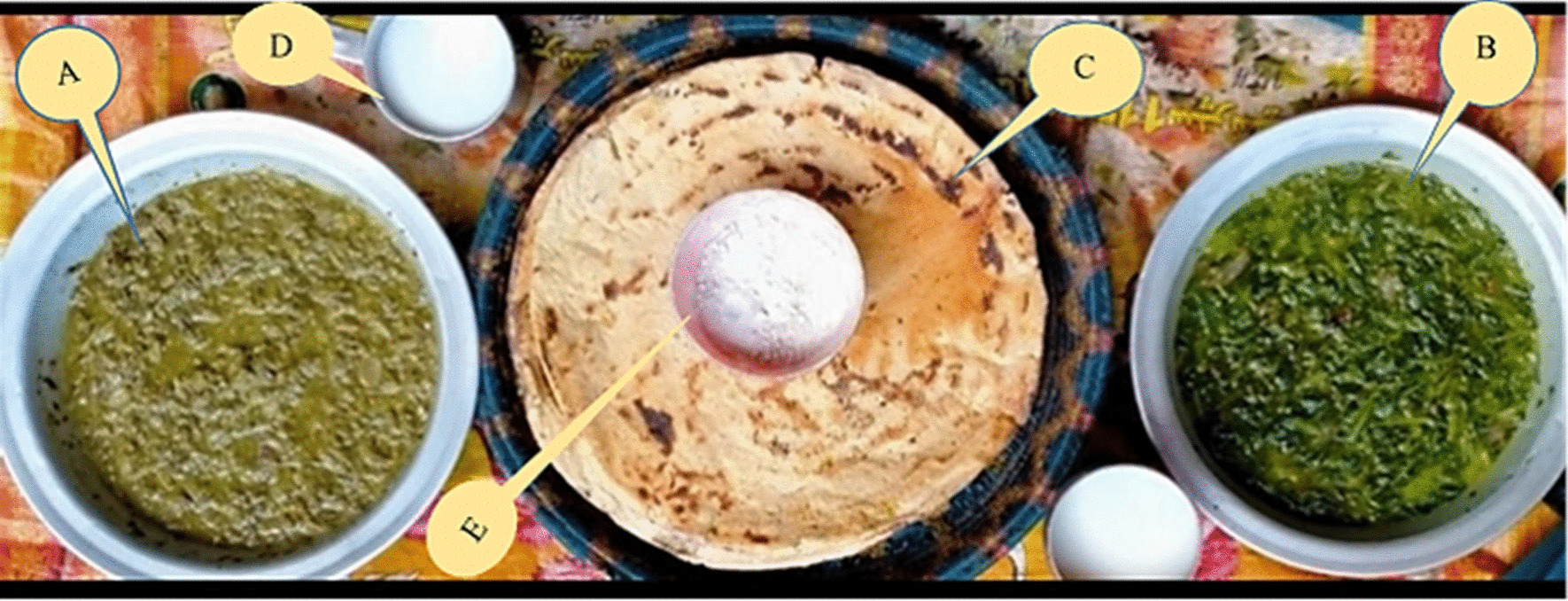
Traditional cuisines of Kohistan Upper. **A** Ghorsa Sha, **B**. Zahoon Sha, **C** Maize bread, **D** buttermilk/Buttermilk, **E** Salt

The local people affirmed that they do not like spicy foods and mostly use salt while cooking foods. They prefer to eat traditional foods prepared from WFPs along with dairy products such as Butter milk, Bilona Ghee, and butter. Among these, Zahoon Sha and Gurasa Sha (Fig. 8) are the most used traditional foods which are quiet famous in Kohistan Upper. Zahoon Sha is prepared from *Urtica dioica* and *Viola pilosa*. Fresh leaves of the plants are collected, then mixed in 2:1 (*U. dioica*: *V. pilosa*) and boiled in water for 10–15 min. Onion and tomato are fried in oil, or Bilona Ghee and mixed with the cooked vegetables and is eaten with bread, Butter milk and common salt. Similarly, Gurasa Sha another traditional cuisine is prepared from *P. oleracea* or *M. longifolia.* Either fresh or dried plant material of any one of these taxa is boiled in Butter milk. When it turns into a dense liquid, then seasoned with onion and tomato priory fried in Bilona Ghee. Another traditional food named Leetee is prepared from maize flour. To prepare Leetee, flour is boiled in water to make a paste. Then, it is added into a mud pot until it cools down. Bilona Ghee is mixed in Leetee and is eaten as a meal. The inhabitants of Kohistan Upper sell dry fruits of *Diospyros lotus* (1–2$, 200–300 rupees/Kg),* Ziziphus jujuba* (0.05 to 1$, 150–200 rupees/Kg), and *Juglans regia* (1.2–2$, 300–400 rupees/Kg) to the local markets, as well as along the roadside of Karakoram Highway (KKH).

**Fig. 9:**
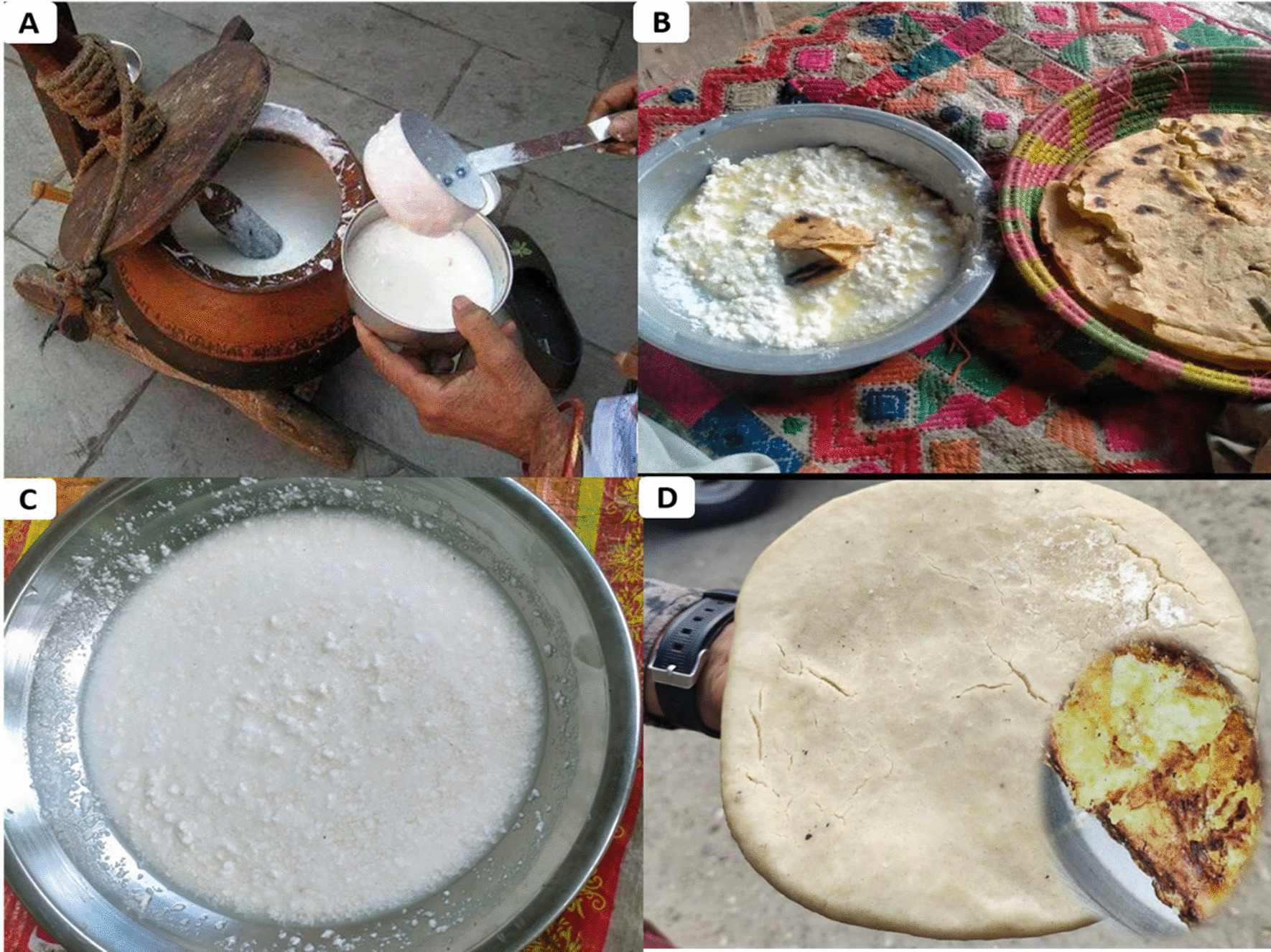
Traditional cuisines of Kohistan Upper. **A** traditional way of making Buttermilk using wooden Chagoor, **B** Bhorus made from Buttermilk, **C** Bak made from Buttermilk, **D** Bagora made from Bhorus

Different types of dairy products, as illustrated in Fig. 9A–D, used in the traditional food system of Kohistan were also recorded. These dairy products are commonly prepared in almost every home and are not consumed directly but used as an important ingredient in plant-based traditional cuisines. Among these, 14 commonly used products are mentioned in Additional file 1: Table S2. Bhorus or Mathar is the most famous dairy product. Bhorus is prepared from Buttermilk. First, buttermilk (Fig. 9A) is made by blending homemade curd in a mud pot with the help of a local wooden device called Chagoor. After vigorous shaking, some amount of water is added into it. This mixture is added into a piece of cloth or bag having minute pores and pressed with hands to remove water. Afterward, this cloth/bag is hung on a tree with wood on the roof for a few days to remove remaining water. At the end a semi-solid material is obtained, which is called Bhorus or Mathar (Fig. 9B). It is eaten raw or with maize bread. Bak is another dairy product. It is prepared from Buttermilk, which is boiled slowly and gradually on fire until converted into a semi-solid material called Bak (Fig. 9C). As mentioned above, traditional cuisines are ingested in a specific way that is locally known as Maddun. To prepare Maddun, first maize bread is crushed in a big mud or silver pot, and then, any one of the prepared dishes like Zahoon Sha, Gurasa Sha, Bhorus or Bak is added into it. Then, locally made Ghee of butter is added and mixed with hands and eaten raw with Buttermilk. These dairy products are prepared by traditional methods using milk of cow, buffalo, goat, or sheep as a main ingredient. Among these, milk cream, curd, buttermilk, butter and Bilona ghee have already been reported in other regions [70–73]. However, inhabitants of Kohistan Upper prepare these products in different ways compared to previous reports. Some other dairy products such as Bhorus, Bagora, Bak, Cholam, Kacha, Gurloo and Poyeen were reported for the first time from this region. Among these dairy products, curd, Buttermilk, Bilona ghee are the most preferred and commonly consumed by local inhabitants. Inhabitants of Kohistan Upper also sold some DPs in local markets to make money. Bogora is sold in local markets (11- 14$, 2500–3000 rupees/Kg), while locally made butter and Bilona Ghee are sold (7- 9$, 1600–2000 rupees/Kg). We have also observed that the meat of different animals is half boiled and dried using salt in sunlight and stored in cotton sacks for further use in winter and snowfall season.

Based on the rich gastronomic practices which we recorded among the studied communities, it has been concluded that it is a sound way to think about the socioeconomic empowerment of local communities through promoting ecological tourism. Ecological tourism will help the local communities to celebrate their cultural food and related local knowledge. They will be able to keep alive their ethnic and food identities and promote their products and innovations and sell them. Initiatives to bring along the traditional food tourism and ecological tourism together will provide an efficient integrated approach for the conservation of food heritage, local biodiversity and traditional pastoral and horticultural practices. Policy makers should pay attention to the revitalization of the local ecological knowledge evolving around the natural resource. Traditional horticultural and pastoral practices could not only be useful in promoting the wellbeing of the community, but also be useful in the production of meat and dairy products.

## Conclusions

The study presents an important stock of local knowledge on wild food plants as well as local dairy products among the studied groups. The area could be recognized as *biocultural refugia.* The idiosyncratic uses of wild food ingredients have revealed that the mountain territory is an important spot for celebrating biocultural diversity as we see that the local communities still retain their distinct food ethnobotanies, especially the Gujjar community whose local plant use is more distinct due to their different human-ecological behavior and is more appealing to be protected. However, there are certain challenges to the local knowledge and biocultural diversity due to the ongoing social change; therefore, appropriate policy measures are required to revitalize the local plant knowledge and to protect the biocultural diversity. Moreover, detailed ethnographic studies should explore the traditional food systems, and related local ecological practices and more specifically the role of women in managing the local ecological practices in order to have a better understanding of the local food systems and the underlying challenges. We suggest that traditional gastronomy should be promoted in food tourism for elevating the socioeconomic status of the local communities.

## Supplementary Information

**Additional file 1: Table S1.** Use repots of wild food plants in different use categories. **Table S2.** Locally made dairy products of Kohistan Upper.

## Data Availability

All the data are presented in tables and figures in the article or as supplementary material. However, for any queries can be directed to the corresponding authors.
