# Supplementary material for: Edible wild plant species used by different linguistic groups of Kohistan Upper Khyber Pakhtunkhwa (KP), Pakistan
Source: J Ethnobiol Ethnomed. 2023 Feb 13;19:6. doi: 10.1186/s13002-023-00577-5 (PMC9923922; doi:10.1186/s13002-023-00577-5)
Supplement: Supplementary file 1 — Additional file 1: Table S1. Use repots of wild food plants in different use categories. Table S2. Locally made dairy products of Kohistan Upper. [file 13002_2023_577_MOESM1_ESM.docx]

**Table S1** Use repots of wild food plants in different use categories

| S. No | Botanical taxa | Use categories | | | | | | |
| --- | --- | --- | --- | --- | --- | --- | --- | --- |
|  |  | Fr. | Veg. | S | FA | CG | T | TURs |
| 1 | *Ajuga integrifolia* |  | 74 |  |  |  |  | 74 |
| 2 | *Allium carolinianum* |  |  | 23 |  |  |  | 23 |
| 3 | *Amaranthus hybridus* |  | 118 |  |  |  |  | 118 |
| 4 | *Amaranthus viridus* |  | 9 |  |  |  |  | 9 |
| 5 | *Barbarea verna* |  | 9 |  |  |  |  | 9 |
| 6 | *Bistorta amplexicaulis* |  | 11 |  |  |  |  | 11 |
| 7 | *Cedrus deodara* |  |  |  |  | 47 |  | 47 |
| 8 | *Celtis caucasica* | 61 |  |  |  |  |  | 61 |
| 9 | *Celtis australis* | 18 |  |  |  |  |  | 18 |
| 10 | *Chenopodium album* |  | 62 |  |  |  |  | 62 |
| 11 | *Cirsium arvense* |  | 42 |  |  |  |  | 42 |
| 12 | *Citrus aurantium* | 23 |  |  |  |  |  | 23 |
| 13 | *Clematis grata* |  | 19 |  |  |  |  | 19 |
| 14 | *Convolvulus arvensis* |  | 56 |  |  |  |  | 56 |
| 15 | *Diospyros lotus* | 98 |  |  |  |  |  | 98 |
| 16 | *Elaeagnus umbellata* | 30 |  |  |  |  |  | 30 |
| 17 | *Ficus palmata* | 82 | 9 |  |  |  |  | 91 |
| 18 | *Duchesnea indica* | 65 |  |  |  |  |  | 65 |
| 19 | *Hypericum perforatum* |  | 13 |  |  |  | 3 | 16 |
| 20 | *Impatiens edgeworthii* |  |  | 29 |  |  |  | 29 |
| 21 | *Indigofera tinctoria* |  |  | 58 |  |  |  | 58 |
| 22 | *Juglans regia* | 81 |  |  |  |  |  | 81 |
| 23 | *Juniperus excelsa* |  |  |  | 9 |  |  | 9 |
| 24 | *Leontopodium himalayanum* |  |  |  |  | 11 |  | 11 |
| 25 | *Mentha longifolia* |  | 106 | 10 |  |  | 9 | 125 |
| 26 | *Morus nigra* | 81 |  |  |  |  |  | 81 |
| 27 | *Myrtus communis* | 43 |  |  |  |  | 11 | 54 |
| 28 | *Nasturtium officinale* |  | 23 |  |  |  |  | 23 |
| 29 | *Olea ferruginea* | 27 |  |  |  |  |  | 27 |
| 30 | *Oxalis corniculata* |  |  | 17 | 82 |  |  | 99 |
| 31 | *Oxyria digyna* |  | 13 |  |  |  |  | 13 |
| 32 | *Parrotiopsis jacquemontiana* | 30 |  |  |  |  |  | 30 |
| 33 | *Pedicularis oederi* |  | 7 |  |  |  |  | 7 |
| 34 | *Persicaria amplexicaulis* |  | 10 |  |  |  |  | 10 |
| 35 | *Pinus gerardiana* | 68 |  |  |  |  |  | 68 |
| 36 | *Pistacia khinjuk* | 13 |  |  |  |  |  | 13 |
| 37 | *Podophyllum hexandrum* | 35 |  |  |  |  |  | 35 |
| 38 | *Portulaca oleracea* |  | 27 |  |  |  |  | 27 |
| 39 | *Primula elliptica* |  |  |  | 11 |  |  | 11 |
| 40 | *Primula macrophylla* |  |  |  | 9 |  |  | 9 |
| 41 | *Prunus armeniaca* | 32 |  |  |  |  |  | 32 |
| 42 | *Prunus persica* | 44 |  |  |  |  |  | 44 |
| 43 | *Punica granatum* | 54 |  |  |  |  |  | 54 |
| 44 | *Pyrus malus* | 64 |  |  |  |  |  | 64 |
| 45 | *Quercus semecarpifolia* | 104 |  |  |  |  |  | 104 |
| 46 | *Ranunculus laetus* |  | 20 |  |  |  |  | 20 |
| 47 | *Rheum australe* |  |  | 32 |  |  |  | 32 |
| 48 | *Rheum emodi* |  |  | 36 |  |  |  | 36 |
| 49 | *Rubus fruticosus* | 65 |  |  |  |  |  | 65 |
| 50 | *Rubus niveus* | 34 |  |  |  |  |  | 34 |
| 51 | *Rumex acetosa* |  | 10 |  |  |  |  | 10 |
| 52 | *Rumex abyssinicus* | 12 | 10 |  |  |  |  | 22 |
| 53 | *Rumex dentatus* |  | 81 |  |  |  |  | 81 |
| 54 | *Rumex hastatus* |  | 44 |  |  |  |  | 44 |
| 55 | *Solanum villosum* | 80 | 21 |  |  |  |  | 101 |
| 56 | *Solanum nigrum* | 35 |  |  |  |  |  | 35 |
| 57 | *Taxus wallichiana* | 12 |  |  |  |  |  | 12 |
| 58 | *Torilis leptophylla* |  | 25 |  |  |  |  | 25 |
| 59 | *Trifolium pratense* |  | 41 |  |  |  |  | 41 |
| 60 | *Urtica dioica* |  | 90 |  |  |  |  | 90 |
| 61 | *Viburnum cotinifolium* | 10 |  |  |  |  |  | 10 |
| 62 | *Viola pilosa* |  | 86 |  |  |  |  | 86 |
| 63 | *Vitis jacquemontii* | 89 |  |  |  |  |  | 89 |
| 64 | *Ziziphus jujuba* | 30 |  |  |  |  |  | 30 |
| Total use reports | | 1420 | 1036 | 205 | 111 | 58 | 23 | 2853 |

Fr. Fruit, Veg. vegetable, S. salad, FA. Flavoring agent, CG. Chewing gum, T. tea, TURs. Total use reports

**Table S2.** Locally made dairy products of Kohistan Upper

| S. No. | Common name | Local name | | | Ingredients | Traditional recipes |
| --- | --- | --- | --- | --- | --- | --- |
|  |  | Kohistani | Shina | Gujari |  |  |
| 1 | Colostrum | Koow |  | Boree | Milky fluid after birth of new ones | Milky fluid of buffalo, cow, or goat is taken instantly after the birth of new baby. This milk is boiled and kept in mud pot at room temperature. After few hours it turns into jell like material locally named as Koow or Boree. It is very delicious product, and everyone likes to eat it. Koow is also distributed freely among the relatives and other people in the village usually up to one week. |
| 2 | Cream | Pervyee |  | Malai | Milk | Fresh milk is boiled, then kept for cooling at room temperature. Top layer of fats is removed with hands in another pot and is eaten with maize or wheat bread. |
| 3 | Curd | Chuk cheer | Mood doodh | Khato dodh | Milk | Fresh milk without boiling is added into a mud pot on daily basis. This mud pot is kept at colder place or near the water channel or in cold air coming from rocks in summer. While in winter mud pot is placed near clay oven. After few days milk is fermented into a semi solid and viscous material called Chuk cheer/Mood doodh/ Khato dodh. Local inhabitants love to eat it with maize bread, especially in breakfast and at lunch time. It is also given to patients and old aged people. |
| 4 | Buttermilk | Goras | Meel | Buttermilk | Curd | Curd or Chuk cheer is added into mud pot and is blended with locally made wooden devise named Chagoor (Figure 5A), while adding little amount of water. Finally, it is converted into thick diluted material called Goras, Meel or Buttermilk. It is most favourite and consumed dairy product in this region. People drink it alone or use it while eating maize bread especially. |
| 5 | Butter | Shasha Gheel |  |  | Curd | During the making of buttermilk (Buttermilk), at final stage small pieces of butter appear on the top, which are pooled together into a separate pot. Fresh butter is eaten raw along with maize or wheat bread, specifically in breakfast. It is also mixed in cooked vegetables, meat and other foods. |
| 6 | Bilona Ghee | Gheel | Ghee | Makhan | Butter | Freshly prepared butter is heat on fire slowly and gradually, to remove remaining parts of buttermilk. After that, it is converted into a yellowish viscous material called ghee, which is stored in mud or silver made container for further use. Ghee is eaten raw with maize of wheat bread. Sometime also mixed in cooked vegetables, meat and other foods. |
| 7 | Fermented Ghee | Chit Gheel | Chut Ghee | Ghee | Bilona Ghee | Freshly prepared Bilona Ghee is also stored for further use. Local inhabitants of KU, pack Bilona ghee in big mud pots or wrap tightly in the bark of *Bitula* tree. Then a deep hole is made in the earth, where packed ghee is stored for many years. This type of ghee, have strong smell and taste that depends how much time it was kept underground. This ghee is very healthy, energetic, and gives special strength to human body. Especially the aged people of the KU love to eat. |
| 8 |  | Bhorus | Bhorus | Mathar | Buttermilk/Buttermilk | The fresh buttermilk/Buttermilk is added into porous cloth bag. It is squeezed forcefully with hands to remove water. After that a viscous/semi-solid material is obtained, which is eaten along with maize or wheat bread. |
| 9 |  | Bagora | Bugrayee | Bagora | Bhorus | Bhorus made of buttermilk is packed in a porous cloth bag and is kept in between heavy stone to press it for 1-2 days. After that it is converted into hard, flat solid beard like material locally named as Bagora or Bugrayee (Figure 5D). |
| 10 |  | Bak | Bak | Bhagoro | To boiled Buttermilk | Bak is made of buttermilk/Buttermilk. To prepare Bak, Buttermilk is boiled slowly and gradually on slow heat. Finally, it is converted into a semisolid material called Bak (Figure 5B). It is eaten raw with salt or mixed with maize bread and ghee and taken as meal. |
| 11 |  | Cholam | Sholoom |  | Butter | Basically, Cholam is a waste of butter, obtained during its conversion into Ghee. During making Biolan ghee, butter is heated again and again, and some impurities settle down in the bottom during heating. Ghee is collected into another pot, while waste at the bottom locally called Cholam or Sholoom. It is very strong and energetic, usually people who work hard eat it with bread and Buttermilk. |
| 12 |  | Kacha | Kacha | Kachee | Cholam and maize flour | Cholam is mixed with maize flour. This mixture is cooked on fire to make Kacha, which is eaten with maize or wheat bread. |
| 13 |  | Gurloo |  | Guloo | Ghee, wheat flour | Wheat flour is mixed in water to make semi paste like material. This material is spread on hot plat/griddle with hand and cooked like a bread with many circles called Gurloo or Guloo. Locally made butter or ghee is added on it and crushed into small pieces with hand and eaten with Buttermilk of milk. |
| 14 |  | Poyeen |  | Letee | Wheat flour, ghee | Poyeen is a bread, which is given as a special gift to bride on her marriage. To make Poyyen, fresh milk, wheat flour, ghee, and sugar/molasses are mixed. This mixture is cooked on fire. After that, it is kept in between two wheat breads and slightly pressed. Then hot ghee is added on it and kept in a round pot to make it like a fat bread. Then it is given to bride as a gift. |
